# Feasibility of Salvage Therapy With High-Dose Bendamustine and Rituximab Based on a Series of Two Cases From a Planned Phase II Trial for Relapsed/Refractory Diffuse Large B-cell Lymphoma

**DOI:** 10.7759/cureus.102788

**Published:** 2026-02-01

**Authors:** Kazunori Murai, Yashushi Hiramatsu, Toru Kiguchi, Nobuhiko Uoshima, Yotaro Tamai, Koji Fukushima, Takuma Furukawa, Toshimi Hoshiko, Ayako Takamori, Tubasa Mitsutake, Noriko Yoshida, Shinya Kimura, Yasuhito Terui

**Affiliations:** 1 Department of Hematology, Iwate Prefectural Central Hospital, Iwate, JPN; 2 Department of Hematology and Oncology, Japanese Red Cross Society Himeji Hospital, Hyogo, JPN; 3 Department of Diabetes, Endocrinology, and Hematology, Dokkyo Medical University Saitama Medical Center, Saitama, JPN; 4 Department of Hematology, Japanese Red Cross Kyoto Daini Hospital, Kyoto, JPN; 5 Department of Hematology, Shonan Kamakura General Hospital, Kamakura, JPN; 6 Hematology, SymBio Pharmaceuticals Limited, Tokyo, JPN; 7 Epidemiology and Public Health, Saga University Hospital, Saga, JPN; 8 Hematology and Oncology, Saga University Hospital, Saga, JPN; 9 Health Policy, Saga University Hospital, Saga, JPN; 10 Health Policy, Hiroshima University Hospital, Hiroshima, JPN; 11 Physical Medicine and Rehabilitation, Saga University Hospital, Saga, JPN; 12 Department of Hematology, Saitama Medical University Hospital, Saitama, JPN

**Keywords:** bendamustine hydrochloride, diffuse, hematopoietic stem cell transplantation, large b-cell, recurrence, rituximab

## Abstract

Background: High-dose chemotherapy combined with autologous hematopoietic stem cell transplantation (HDC/AHSCT) is an important option to improve the prognosis of relapsed or refractory diffuse large B-cell lymphoma (R/R DLBCL). Combination therapy of bendamustine and rituximab (BR120: 120 mg/m^2^/day bendamustine for two consecutive days, with 375 mg/m^2^ rituximab) has shown good efficacy against R/R DLBCL. However, its myelosuppressive effects may impair subsequent hematopoietic stem cell (HSC) mobilization and collection.

Methods: This study involved patients treated at two institutions: the Department of Hematology, Iwate Prefectural Central Hospital (Iwate, Japan), and the Department of Hematology and Oncology, Japanese Red Cross Society Himeji Hospital (Hyogo, Japan). To evaluate the feasibility of HSC mobilization and the efficacy and safety of BR120 combined with G-CSF and plerixafor, we conducted a multicenter, open-label, phase II clinical study of BR120 salvage therapy in patients with R/R DLBCL indicated for HDC/AHSCT. Patients underwent HSC collection and continued BR120 therapy for up to four cycles. The expected number of patients enrolled in the 11 participating institutions is 20.

Results: Only two patients were enrolled in the study. However, both also achieved a target harvest of 2×10^6^ cells/kg of CD34^+^ cells within 2 days of apheresis. The mobilization-adjusted response rate was 100%. Grade 3-4 adverse events included lymphopenia in both patients.

Conclusion: We could achieve successful mobilization and stem cell collection with BR120 salvage therapy in two cases.

## Introduction

Diffuse large B-cell lymphoma (DLBCL) is a form of malignant lymphoma categorized as a mature B-cell tumor by the World Health Organization (WHO) disease classification. The treatment of DLBCL has made dramatic progress in recent years through clinical and genetic research. Among them, R-CHOP, Pola+R-CHP, and others have been recommended as standard care for the primary treatment of untreated DLBCL [1,2]. Although clinical outcomes have improved significantly, the treatment of relapsed or refractory DLBCL (R/R DLBCL) remains a clinical issue to improve the prognosis. In recent years, chimeric antigen receptor-T-cell (CAR-T) therapy has shown a highly durable response for R/R DLBCL [3-5]. High-dose chemotherapy with autologous hematopoietic stem cell transplantation (HDC/AHSCT) is an important second-line treatment option for DLBCL that relapses or becomes refractory after 12 months [6]. However, the superiority of salvage chemotherapy for R/R DLBCL remains unclear [7,8].

Bendamustine hydrochloride was synthesized in the early 1960s by Jenapharm in East Germany and has a nitrogen mustard group and a purine-like benzimidazole ring in its chemical structure. It combines the properties of both alkylating agents and antimetabolites [9]. Standard BR regimens (BR90) commonly use bendamustine at 90 mg/m²/day for two days, with rituximab 375 mg/m^2^ for follicular lymphoma or mantle cell lymphoma [10]. The newly approved combination of rituximab (375 mg/m^2^ intravenous infusion) and bendamustine hydrochloride (120 mg/m^2^/day intravenous infusion for two consecutive days) (BR120) for R/R DLBCL showed good clinical efficacy [11-13]. The main side effects of BR120 include myelosuppression, pneumonia, sepsis, and interstitial pneumonia. Alopecia, which is observed with platinum-containing therapies, is rare and occurs in less than 10% of cases.

However, there are concerns that myelosuppression, a characteristic side effect of BR120, may affect hematopoietic stem cell (HSC) harvesting after salvage chemotherapy [14]. An important clinical question is whether BR120 therapy should be administered as salvage chemotherapy in patients with indications for transplantation. The present study was designed to assess the efficacy and safety of BR120 in combination with granulocyte colony-stimulating factor (G-CSF) and plerixafor for HSC mobilization and harvesting.

The present study was designed to evaluate HSC mobilization, as well as the efficacy and safety of BR120 combined with G-CSF and plerixafor for stem cell mobilization and collection.

## Materials and methods

A multicenter, open-label, single-arm, phase II clinical study was conducted in patients with R/R DLBCL who were indicated for HDC/AHSCT. This study involved patients treated at two institutions: the Department of Hematology, Iwate Prefectural Central Hospital (Iwate, Japan), and the Department of Hematology and Oncology, Japanese Red Cross Society Himeji Hospital (Hyogo, Japan).

Patient eligibility

We recruited patients between 20 and 65 years old with DLBCL who met all of the following inclusion criteria: (1) histopathologically confirmed DLBCL based on the 2017 WHO Classification of Tumors of Hematopoietic and Lymphoid Tissues, 4th Edition [15] (in principle, the most recent histopathological specimen was used to diagnose the disease in individual patients); (2) tumor cell positivity for CD20 demonstrated by immunohistochemistry or flow cytometry (≥ 20%); (3) experienced relapse or refractory after primary treatment with standard R-CHOP or a similar regimen such as R-CHOEP (rituximab, cyclophosphamide, hydroxydaunorubicin, etoposide, vincristine and prednisolone) or R-THP-COP (rituximab, pirarubicin, cyclophosphamide, vincristine, and prednisolone); (4) patients deemed candidates for HDC/AHSCT by the treating physician; (5) presence of measurable lesions >1.5 cm in diameter confirmed by computed tomography (CT); (6) patients expected to survive for at least three months; (7) a period of at least 12 months after prior therapy until relapse or refractory; (8) performance status (PS) of 0 to 2 (Eastern Cooperative Oncology Group Performance Status criteria) with a preserved major organ function; and (9) provided their written informed consent to participate in this study. The exclusion criteria were transformed lymphoma, evidence of central nervous system involvement, uncontrolled infection, and rituximab resistance or intolerance.

Eleven centers participated in this multicenter, open-label, unblinded, single-arm, phase II clinical trial (jRCTs071210121), with patient enrollment from January 26, 2022, to March 31, 2023. In accordance with the Declaration of Helsinki (revised 2013), written informed consent was obtained from all patients using an informed consent form approved by the Saga University Certified Clinical Research Review Board (CRB) (approval number CRB7180010) and the administrator at each study site. Although the planned sample size was 20 patients, only two patients were enrolled in the study. The sample size was estimated based on experience in conducting clinical trials from the perspective of trial feasibility and safety assessment, as it was difficult to calculate the sample size with high accuracy as a preliminary design.

Study design 

The duration of the study was from obtaining consent to the completion of four cycles of BR120 therapy. In principle, one cycle lasted 21 days. If complete remission (CR) or partial remission (PR) was achieved after two cycles, the patient proceeded to the collection of HSCs while receiving up to four cycles. If no response was achieved after two cycles, study participation was terminated, and if the patient continued BR120 therapy after study termination, six cycles were allowed as the maximum. The antitumor response was evaluated based on the revised response criteria for malignant lymphoma (2007) [16] and classified into CR, PR, SD, and relapsed disease /PD based on the comprehensive response criteria.

BR120 salvage therapy and apheresis

**Figure 1:**
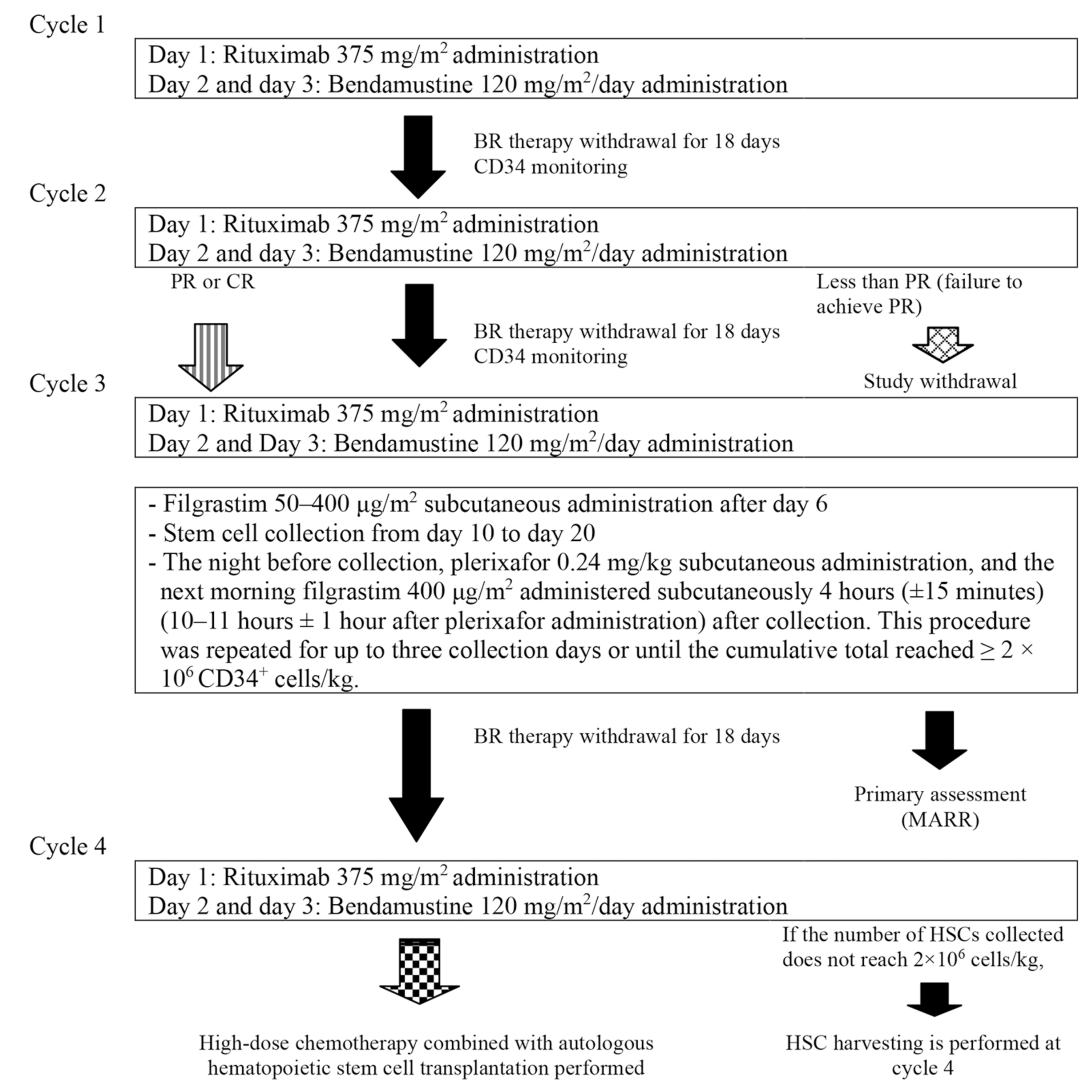
Overview of study procedures BR120 therapy, bendamustine hydrochloride and rituximab therapy; PR, partial remission; HSCs, hematopoietic stem cells; MARR, mobilization-adjusted response rate

The study patients received intravenous bendamustine and rituximab in a 21-day cycle for up to four cycles. In each cycle, short-acting G-CSF (filgrastim) was administered for neutropenia after BR120 salvage therapy, but the use of sustained G-CSF was not allowed. Cycle delays up to 14 days were permitted (maximum cycle length 35 days). Each cycle consisted of rituximab 375 mg/m^2^ intravenous infusion on day 1 and bendamustine 120 mg/m^2^/day for 2 consecutive days on days 2 and 3, with an interval period of at least 18 days (Figure 1).

In cycle 3, after BR120 therapy, G-CSF (50-400 μg/m^2^) was administered subcutaneously once daily from day 6. Based on the results of peripheral blood CD34⁺ cell count (≥20/mm^3^) and/or white blood cell count (≥5000/mm^3^) monitoring, plerixafor 0.24 mg/kg was administered subcutaneously the night before HSC collection. Apheresis was performed four hours (±15 minutes) after G-CSF administration, the following morning (i.e., 10-11 ± 1 h after plerixafor administration). This procedure was repeated up to three times or until ≥2 × 10⁶ CD34⁺ cells/kg were collected. If a sufficient number of cells was not obtained after cycle 3, the same procedure was performed after cycle 4. Apheresis was performed from approximately day 10 to day 20 at the discretion of the principal investigator or sub-investigator, based on the results of CD34^+^ cell monitoring.

In addition, the following laboratory test results were confirmed three weeks after the start of each cycle: neutrophil count ≥1,000/mm^3^; platelet count ≥75,000/mm^3^; total bilirubin <2.0 mg/dL; serum creatinine <2.0 mg/dL; and <grade 2 nonhematologic toxicity or less (based on NCI-CTCAE Version 5) [17]. If not, the next cycle could be postponed for up to two weeks. In addition, from cycle 2 onward, if adverse events occurred during the previous cycle period corresponding to <500/mm^3^ or remained<1,000/mm^3^ lasting at least two weeks in neutrophil count, <75,000/mm^3^ in platelet count, or nonhematologic toxicity of grade ≥3, a reduction in the bendamustine dose (levels2 and 3 of 90 and 60 mg/m^2^/day, respectively) was specified.

Study assessments and endpoints

The primary endpoint was the response rate (mobilization-adjusted response rate; MARR, response rate corrected for the percentage of patients who successfully harvested HSCs after three cycles of salvage chemotherapy) adjusted for the percentage of patients who had successful HSC harvesting after three cycles of salvage chemotherapy. Thus, MARR was evaluated as the overall response rate (ORR) with the cumulative total reached ≥ 2×10^6^ CD34^+^cells/kg.

The secondary endpoints were the CR rate according to the Revised Response Criteria for Malignant Lymphoma after two cycles of salvage chemotherapy, ORR after two cycles of salvage chemotherapy, percentage of patients who achieved the target CD34^+^ cell collection rate of 2×10^6^ cells/kg within 2 days of apheresis, and number of apheresis days required to collect at least 2×10^6^ cells/kg of CD34^+^ cells. Using histopathological specimens subjected to pathological diagnosis, subtype classification, and minimal residual disease (MRD) within the harvested stem cell collection were also considered exploratory endpoints. MRD was detected using clonoSEQ®, an NGS-based MRD detection method for B-cell lymphoma developed by Adaptive Biotechnologies (test facility: RIKEN GENESIS Co., Ltd., Tokyo, Japan).

CT scanning was performed as an imaging test, and fluorodeoxyglucose-positron emission tomography (FDG-PET; PET-CT when possible) at screening and during cycle 2 was also performed, which covered an area sufficient to include lesions from the head to the pelvic region (including the orbit on the head side and inguinal lymph nodes on the legs) and images from two- or three-dimensional collections (MIP images, fusion images, and cross-sectional images in each direction).

For safety, adverse events due to subjective symptoms and objective findings were recorded, and general blood, blood biochemical, and immunological tests were performed. The severity of adverse events was defined according to the Common Terminology Criteria for Adverse Events v5.0, a Japanese translation of the JCOG version (CTCAE v5.0-JCOG) [17]. For items not listed in the CTCAE v5.0-JCOG, severity was judged using the prescribed severity criteria. Narrative terms for adverse events were coded according to the Medical Dictionary for Regulatory Activities/Japanese (MedDRA/J) version 24.1.

Statistical analyses

The MARR, CR rate, and ORR were calculated using the full analysis set (FAS). The percentage of patients achieving the target CD34^+^ cell collection rate and the number of days of apheresis were also determined.

## Results

Patients

**Figure 2:**
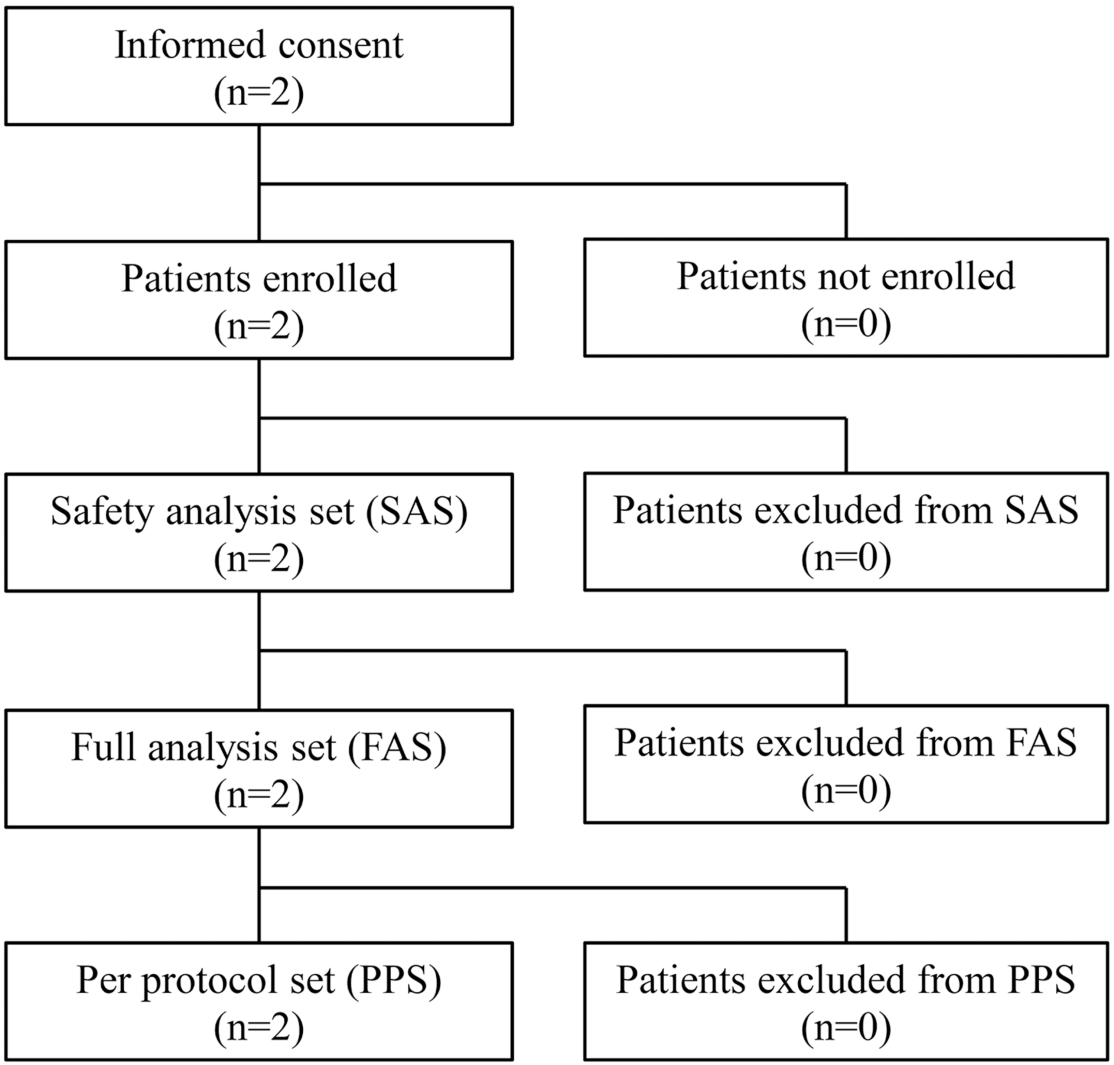
Patient dispositions

The study was originally designed to enroll 20 patients, but only two were able to participate. These patients (1 and 2) were men, 56 and 61 years old, respectively (Figure 2). 

Both patients were responders (CR/PR) to previous treatment with relapse after one regimen, with stage IV clinical disease based on the Ann Arbor classification. The genetic subtype of Patient 2 was EZB (Tables 1, 2). 

**Table 1:** Patient characteristics ECOG, eastern cooperative oncology group; DLBCL, diffuse large B-cell lymphoma; NOS, not otherwise specified; GCB, germinal center B-cell; LDH, lactate dehydrogenase; R-THP-COP, rituximab +pirarubicin +cyclophosphamide +vincristine sulfate +prednisone; R-CHOP, rituximab + cyclophosphamide +vincristine sulfate +prednisone

Item		Patient 1	Patient 2
Attribute	Sex	Male	Male
	Age (years)	56	61
	Height (cm)	166.7	172.7
	Body weight (kg)	70	69
	Body surface area (m^2^)	1.74	1.77
Status	Medical history	None	Hernia, retinal detachment
	Compilations	Asthma	Gout
	EOCG performance score	0	2
Diagnosis and genetic abnormality	Histopathological diagnosis	DLBCL	DLBCL
	Central Pathology Review (WHO classification)	NOS	NOS
	Period from diagnosis to recurrence (months)	62.5	28.6
	Hans algorithm (cell of origin)	Non-GCB	GCB
	Genetic subtype	Unclassified	EZB
	*MYC*/*BCL2*/*BCL6* reconfiguration	None	*MYC* 8q24 translocation */ BCL2* 18q21 translocation
	Clinical stage (Ann Arbor classification)	IV	IV
Previous medication	Treatment history	Yes	Yes
	Number of treatment regimens	1	1
	Treatment regimen	R-THP-COP	R-CHOP
	Response to treatment	Responder	Responder
Disease risk	LDH	Below normal upper limit (216 U/L)	High value (301 U/L)
	Number of nodal lesions	>5 or more	<5
	Number of extranodal lesions	<2	>2 or more
	Bone marrow involvement	Positive	Negative
	The international prognostic index (IPI) risk category	Low	High

**Table 2:** Individual patient characteristics DLBCL, diffuse large B-cell lymphoma; GCB, germinal center B-cell-like type; EZB, based on EZH2 mutations and BCL2 translocations; ECOG, Eastern Cooperative Oncology Group. ^1)^Classification by the National Institutes of Health (NIH). ^2)^ The prior therapy for each patient was R-THP-COP (rituximab + pirarubicin + cyclophosphamide + vincristine sulfate + prednisone); or R-CHOP (rituximab + cyclophosphamide + vincristine sulfate + prednisone). ^3)^Patients in whom an excellent response to 1 or more previous treatments (CR or PR) were defined as “responders” and other patients were defined as “nonresponders.” ^4)^Total number of target lesions and nontarget lesions at nodes classified as CT type or nontarget CT type. ^5)^Total number of target lesions at nodes classified as CT type or nontarget CT type and bone marrow infiltration (if positive). ^6)^Classified according to the number of the following relevant prognostic factors: age, 61 years or older; LDH, above the upper limit of normal; PS, 2–4; clinical stage, III or IV; number of extranodal lesions, two or more.

Variable		n=2
Sex (male), n		2
Age (years), range, n	50–59 / 60–65	1 / 1
Diagnosis	DLBCL	2
Pathology diagnosis at each medical institute, Hans algorithm (cell of origin)	GCB / Non-GCB	1 / 1
Genetic subtypes^1)^	EZB / Unclassified	1 / 1
Clinical stage (Ann Arbor staging)	IV	2
History of prior treatment (yes), ^2) ^n		2
Number of prior treatment regimens, n	1 regimen	2
Response to prior treatment,^3)^ n	No / Yes	0 / 2
History of autologous hematopoietic stem cell transplantation	No / Yes	2 / 0
History of radiation therapy treatment	No / Yes	2 / 0
ECOG performance status	0 / 2	1 / 1
Number of nodal lesions^4)^	< 5 / ≥ 5	1 / 1
Number of extranodal lesions^5)^	< 2 / ≥ 2	1 / 1
Bone marrow infiltrate	Positive / Negative	2 / 0
International Prognostic Index (IPI) risk category^6)^	Low / High	1 / 1

Owing to the limited number of enrolled patients, the efficacy assessment based on the planned statistical method could not be fully validated. Therefore, the clinical findings of each patient and the apheresis implementation status were evaluated in detail.

ORR after two cycles of BR120 therapy

In the overall efficacy assessment after two cycles of BR120 therapy, patients 1 and 2 achieved CR and PR, respectively. The CR and ORR rates were 50% and 100% (2/2), respectively (Table 3). 

**Table 3:** Antitumor effect and apheresis outcome after two cycles of BR120 therapy ORR, overall response rate; MARR, mobilization-adjusted response rate ^1)^Tumor shrinkage (increase) rate = Sum of two-way products (SPD) at evaluation of antitumor effects – SPD at screening ÷ SPD at screening × 100. ^2)^Determined according to Revised Response Criteria for Malignant Lymphoma (2007). ^3)^Percentage of patients achieving complete remission (CR) or partial remission (PR) after cycle 2. ^4)^If HSC collection was successfully completed after cycle 3 with determination of CR or PR after cycle 2, the patient was considered a responder. *For apheresis, G-CSF 75 μg was administered subcutaneously once a day for 3 days and 675 μg for 6 days from 3 days after the final administration of bendamustine (day 6), and plelixalfor 16.08 mg was administered subcutaneously on the day before collection (day 13 and day 14). **For apheresis, G-CSF 600 μg was administered subcutaneously once a day for 6 days from 3 days after the final administration of bendamustine (day 6), and plelixalfor 15.00 mg was administered subcutaneously on the day before collection (day 10 and 11).

	Patient 1	Patient 2
Total number of CD34^+^ cells collected (cells/kg)	3.01 × 10^6*^	2.48 × 10^6**^
Number of days of apheresis	2	2
Tumor shrinkage rate after cycle 2^1)^	100.0%	95.70%
Overall efficacy assessment^2)^	CR	PR
ORR^3)^	100% (2/2)	
MARR^4)^	100% (2/2)	

The sum of 2-way products (cm^2^) for the greatest diameter of target lesions was reduced from 23.56 at screening to 0.00 after 2 cycles (day 52 from the BR120 therapy start date) in Patient 1 and from 157.23 to 6.80 (day 36 from the BR120 therapy start date) in Patient 2. However, Patient 2 discontinued the study after completing three cycles of BR120 therapy and apheresis due to exacerbation of the underlying disease. The overall efficacy assessment at the time of study discontinuation indicated PD.

Apheresis

In cycle 3, filgrastim was administered from day 6, and the dose of filgrastim at the time of apheresis for each patient was 675 μg on days 13 and 14 for Patient 1, and 600 μg on days 10 and 11 for Patient 2. The peripheral blood CD34^+^ cell counts were 12/μL on day 13, 21/μL on day 14 for Patient 1, and 23/μL on day 11 for Patient 2. In both patients, apheresis was performed after three cycles of BR120 therapy. The total number of cells collected over two days was 3.01×10^6^ cells/kg for Patient 1 and 2.48×10^6^ cells/kg for Patient 2. Both patients were able to achieve the target number of CD34^+^ cells (≥2×10^6^ cells/kg) within two days (Tables 3, 4). Both patients were positive for MRD; Patient 1 had 7.07×10^6^ cells and Patient 2 had 8.37×10^8^ cells. 

**Table 4:** Details of peripheral blood stem cell collection after two cycles of BR120 therapy *Value measured the next day (C3D12). NE, not evaluable; C, cycle; D, number of days from the first treatment in the cycle as day 1; WBC, white blood cell count; PBSCH, peripheral blood stem cell harvest; PB, peripheral blood; HPC, hematopoietic progenitor cell

	Period	WBC (/µL)	HPC in PB (/µL)	CD34 in PB (/µL)	PBSCH (CD34) (× 10^6^ cells/kg)	Total PBSCH (CD34) (× 10^6^ cells/kg)
Patient 1	C3D13	11160	14	12	0.81	
	C3D14	16290	20	21	2.20	3.01
Patient 2	C3D10	N.E.	NE	NE	1.60	
	C3D11	44900*	NE	23	0.88	2.48

**Table 5:** Adverse events *MedDRA Japanese translation (MedDRA/J) version 24.1 (Japanese Maintenance Organization (JMO))

Preferred terms*	Grade 1–2	Grade 3–4	Total
	n	n	n
Anemia	1	0	1
Nausea	1	0	1
Immunodeficiency	1	0	1
Cytomegalovirus viraemia	1	0	1
CD4 lymphocyte count decreased	0	1	1
Immunoglobulin decreased	1	0	1
Lymphocyte count decreased	0	2	2
Lymphocyte count increased	1	0	1
Neutrophil count decreased	2	0	2
Platelet count decreased	1	1	2
T-lymphocyte count decreased	1	0	1
White blood cell count decreased	1	1	2
Arthralgia	1	0	1
Upper respiratory tract inflammation	1	0	1
Pruritus	1	0	1

Thus, the MARR (response rate corrected for the percentage of patients successfully harvesting HSCs after three cycles of BR120 as salvage chemotherapy) was 100% (2/2), although the evaluation of confidence intervals was limited owing to the limited number of patients (Table 3).

Safety

The overall incidence of adverse events is presented in Table 5.

Adverse events of grades 3-4 were decreased lymphocyte count in both patients, decreased CD4 lymphocyte count, decreased white blood cell count, and decreased platelet count in one patient each.

Ten adverse events were reported in Patient 1. Grade 3-4 adverse events included lymphocyte count decreased (two events), CD4 lymphocyte count decreased to grade 4, white blood cell count decreased (two events, grade 3), and platelet count decreased (grade 3). In addition, there was one event each of neutrophil count decreased (grade 2), cytomegalovirus (CMV) viremia (grade 2), immunodeficiency (grade 1), and T-lymphocyte count decreased (grade 1). Valganciclovir hydrochloride was used in combination with G-CSF in cycles 1 and 4 for CMV viremia.

**Figure 3:**
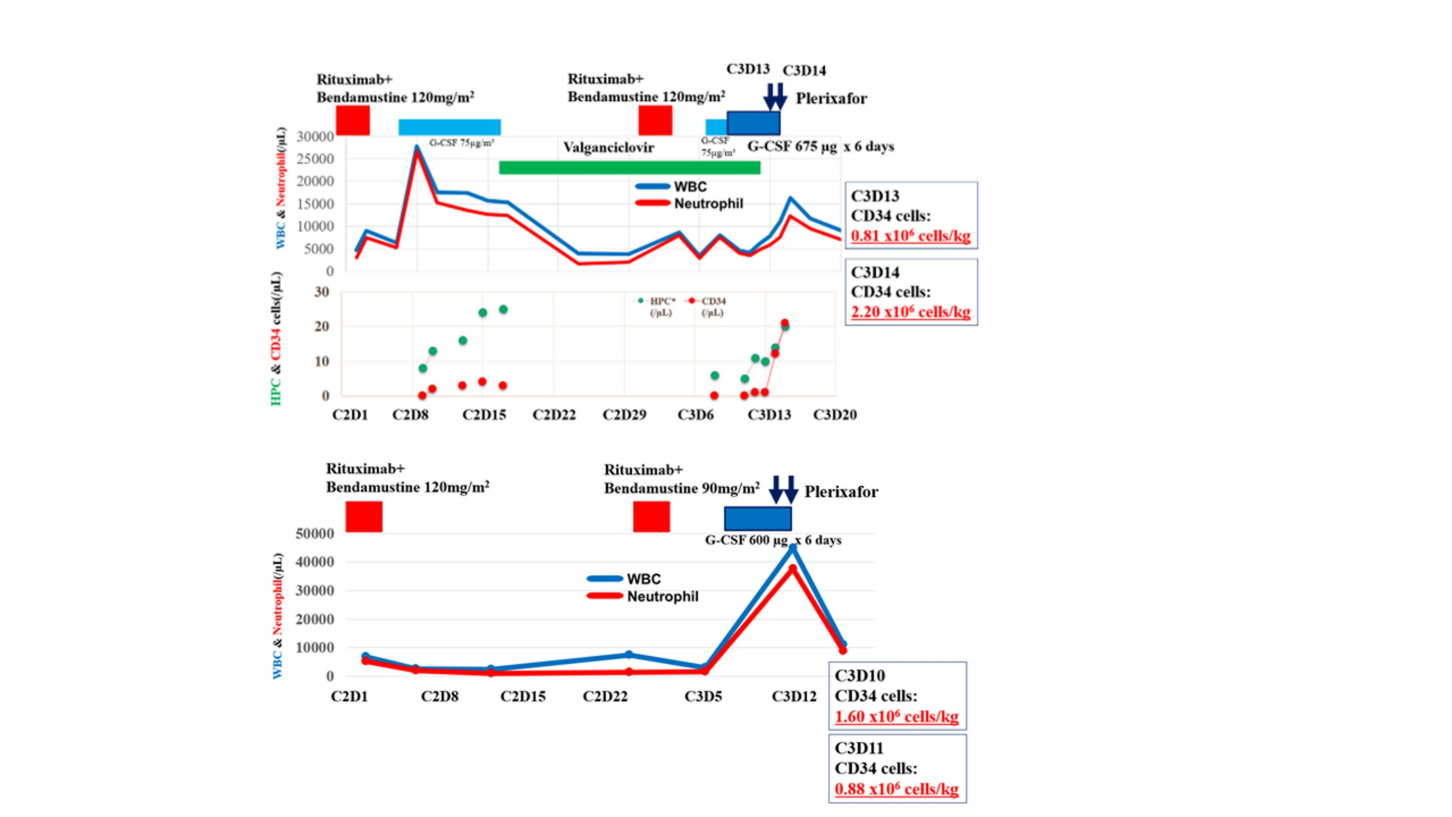
Laboratory values measured prior to apheresis A and B show the progress of patient 1 and patient 2, respectively. R-BEN120, bendamustine hydrochloride 120 mg/m^2^ for two days and rituximab; R-BEN 90, bendamustine hydrochloride 90 mg/m^2^ for two days and rituximab; G-CSF, filgrastim (genetical recombination); WBC, white blood cell count; HPC, hematopoietic progenitor cell; C3D10, cycle 3 day 10; C3D11, cycle 3 day 11; C3D13, cycle 3 day 13; C3D14: cycle 3 day 14 Image created by the authors with Microsoft Word, Microsoft Excel, and Microsoft PowerPoint (Microsoft Corp., USA)

Thirteen adverse events were reported in Patient 2. Grade 3-4 adverse events include decreased lymphocyte count (two events of grade 4 and one event of grade 3). Other adverse events included nausea, upper respiratory tract inflammation, pruritus, a decreased white blood cell count, a decreased neutrophil count, an increased lymphocyte count, a decreased platelet count, anemia (each grade 2), decreased immunoglobulin levels, and arthralgia (each grade 1). A decrease in the platelet count was observed in cycle 2, and the dose of bendamustine was reduced in cycle 3. The time course of white blood cell and neutrophil counts, bendamustine dose, rituximab dose, and apheresis transition is shown in Figure 3. 

## Discussion

This study evaluated the mobilization, clinical efficacy, and safety of BR120 in combination with G-CSF and plerixafor in R/R DLBCL. Although only two patients were enrolled (the planned sample size was 20 patients), both achieved an adequate volume of HSC mobilization. The most common adverse event was myelosuppression with grade 3 lymphopenia and CMV viremia in one of the two patients.

Over the past few years, CAR-T therapy has also become a treatment option for R/R DLBCL that fails to achieve CR with primary treatment or relapse within 12 months. The long-term outcomes of CAR-T therapy and ASCT were compared with those of R/R DLBCL patients. Axicabtagene ciloleucel and lisocabtagene maraleucel established the superiority of CAR-T therapy over the standard of care for primary refractory or early relapse DLBCL [18,19]. Single-center data from China reported that CAR-T therapy demonstrated superior efficacy compared with ASCT [20]. By contrast, the BELINDA trial failed to demonstrate the superiority of tisagenlecleucel over ASCT [21]. Based on an analysis of the Center for International Blood and Marrow Transplant Research registry database, a lower relapse rate and a superior overall survival were observed in patients who underwent ASCT than in those who underwent CAR-T [22]. In addition, a systematic review including 16 studies and 3,484 patients showed that while CAR-T therapy was associated with a higher ORR, ASCT was more effective in terms of the one- and two-year overall survival [23]. In other words, high-dose chemotherapy followed by ASCT may be an effective treatment option for patients younger than 65 years old who relapse 1 year or later after remission and respond (CR + PR) to salvage therapy [24].

However, whether or not a specific salvage regimen is superior in this context remains unclear. Salvage regimens such as R-ICE (rituximab, ifosfamide, carboplatin, etoposide), R-DHAP (rituximab, dexamethasone, Ara-C, cisplatin), and R-GDP (rituximab, gemcitabine, dexamethasone, and cisplatin) are known. In the CORAL study [7], R-DHAP or R-ICE therapy was followed by ASCT, and the efficacies of the two regimens were comparable. The prospective mobilization failure rate was reported to be approximately 10% with both regimens. In the Canadian LY.12 study [8], R-GDP or R-DHAP was followed by ASCT, and the efficacy was also similar. Similarly, the mobilization success rate following R-GDP therapy is reported to be 87.9%. However, which combination chemotherapy regimens are superior to salvage therapies remains unclear.

BR120 as a salvage therapy for R/R DLBCL has also been reported. In a phase II trial from the U.S., the ORR was 45.8% (CR, 15.3%; PR, 30.5%), the median DOR was 17.3 months, and the median progression-free survival (PFS) was 3.6 months [12]. In the Japanese Phase III trial, the ORR and CR rates were 76.3% and 47.4%, respectively, with a median follow-up of 19.5 months, including long-term follow-up, and the median PFS was 11.9 months. BR120 for DLBCL is a salvage therapy that is worth investigating [11]. Bendamustine is highly myelosuppressive and may impair peripheral blood stem cell (PBSC) mobilization. There are no reports of PBSC collection using BR120. This presents a challenge for patients who undergo ASCT. Furthermore, Pola-BR therapy, which combines polatuzumab plus vedotin, an antibody-drug conjugate targeting CD79b, with rituximab plus bendamustine, has been reported to have excellent efficacy and safety in R/R DLBCL [25,26]. These salvage therapies, in which bendamustine is used, are generally administered to patients who are ineligible for ASCT. However, there are scattered reports that Pola-BR plus plerixafor can also be used for PBSC collection [27,28]. In some cases, ASCT may be considered during salvage therapy involving bendamustine.

In the present study, both patients achieved the minimum target cell yield (≥2 × 10^6^ CD34^+^ cells/kg), and the MARR was 100%. The protocol indicated that rituximab should be administered on day 1 of the third course and bendamustine on days 2 and 3, followed by filgrastim from day 6, with stem cells harvested on days 13-20. In addition, plerixafor was administered at a dose of 0.24 mg/kg the night before harvesting. Generally, mobilization strategies include collection during the bone marrow recovery phase after chemotherapy, G-CSF alone, and G-CSF in combination with plerixafor [29]. It is difficult to determine the collection date using this method. Rather, it may be more convenient to collect HSCs using G-CSF and plerixafor after adequate recovery from myelosuppression with BR120.

Both patients in this study experienced grade 3 or 4 hematologic adverse events that required infection management and prophylaxis. One patient (Patient 2) developed thrombocytopenia, necessitating a reduction in bendamustine dose. The other patient (Patient 1) developed CMV viremia during cycle 2, which resolved with valganciclovir hydrochloride administration. Despite the myelosuppressive effects of BR120 therapy, apheresis was successfully performed in both patients. However, careful management and infection prevention, including the monitoring of viral reactivation, are essential, indicating that BR120 therapy should be administered under appropriate supportive care. Under appropriate management, the toxicity was consistent with the known safety profile of bendamustine and did not interfere with the success of collection.

Several limitations associated with the present study warrant mention. First, although the planned sample size was 20 patients, only 2 were enrolled. Second, the purpose of this study was to analyze stem cell mobilization after BR120. However, in one of the two patients, the dose of bendamustine had to be reduced because of thrombocytopenia. The reasons for not reaching the target number of cases may be due to the historical background of the study, including the coincidence of the start of the study and the COVID-19 pandemic infection, and the fact that the use of bendamustine hydrochloride suppresses lymphocytes, making it difficult to implement gene-modified T-cell therapy and bispecific antibody therapy.

Salvage chemotherapy for R/R DLBCL currently lacks a standard treatment, leading to selection based on the treating physician's consideration of the patient's overall condition and disease status. Although the number of cases is limited to 2, both cases achieved the predefined mobilization target (≥2 × 10⁶ CD34⁺ cells/kg), confirming the feasibility of this approach using BR120 for salvage therapy.

## Conclusions

Although these preliminary results are based on two cases and are limited in scope, this is the first clinical trial report to demonstrate the feasibility of HSC collection using BR120 as salvage therapy. The details of this mobilization protocol, including the timing and thresholds for CD34⁺ cell monitoring in this trial, will provide useful practical insights for clinicians attempting stem cell collection in similar clinical situations.

However, to resolve the clinical question of whether BR120 therapy should be selected as salvage chemotherapy for transplant-eligible cases, large-scale comparative clinical trials are required to confirm its efficacy and safety. ASCT remains a valuable treatment option for all patients, not just those who have difficulty accessing CAR-T therapy. Further studies are warranted to establish more flexible and effective mobilization protocols.
